# Serum proinsulin levels as peripheral blood biomarkers in patients with cognitive impairment

**DOI:** 10.1038/s41598-023-49479-2

**Published:** 2023-12-17

**Authors:** Abhinbhen W. Saraya, Chavit Tunvirachaisakul, Chanikarn Sonpee, Panticha Katasrila, Tanyares Sathaporn, Supatporn Tepmongkol, Sookjareon Tangwongchai

**Affiliations:** 1 Division of Neurology, Department of Medicine, Faculty of Medicine, Chulalongkorn University, Bangkok, Thailand; 2 Thai Red Cross EID-Health Science Center, King Chulalongkorn Memorial Hospital-The Thai Red Cross Society, Bangkok, Thailand; 3 Cognitive Impairment and Dementia Research Unit, Faculty of Medicine, Chulalongkorn University, Bangkok, Thailand; 4 Department of Psychiatry, Faculty of Medicine, Chulalongkorn University, Bangkok, Thailand; 5 Department of Radiology, Faculty of Medicine, Chulalongkorn University, Bangkok, Thailand

**Keywords:** Cognitive neuroscience, Molecular neuroscience

## Abstract

Insulin has long been associated with dementia. Insulin affecting the clearance of amyloid-β peptide and phosphorylation of tau in the CNS. Proinsulin is a precursor of insulin and its elevated serum levels are associated with peripheral insulin resistance that may reduce brain insulin levels. Our study aimed to assess differences in serum proinsulin levels between normal and cognitive impairment groups. Prospective recruitment of elderly participants was initiated from October 2019 to September 2023. Patients were divided into “cognitive impairment” and “normal cognition” group. All participants had blood drawn and serum proinsulin was measured at baseline and 12 months. Neurocognitive testing was performed every 6 months. A total of 121 participants were recruited. Seventy-seven were in the normal cognition group and 44 in the cognitive impairment group. The glycemic control and prevalence of diabetes type 2 was similar between groups. Baseline serum proinsulin levels were higher in the cognitively impaired group compared to the normal group at baseline (p = 0.019) and correlated with worse cognitive scores. We identified cognitive status, age, and BMI as potential factors associated with variations in baseline proinsulin levels. Given the complex interplay between insulin and dementia pathogenesis, serum biomarkers related to insulin metabolism may exhibit abnormalities in cognitive impaired patients. Here we present the proinsulin levels in individuals with normal cognitive function versus those with cognitive impairment and found a significant difference. This observation may help identifying non-diabetic patients suitable for treatment with novel AD drugs that related to insulin pathway.

## Introduction

Dementia is a prevalent health predicament among the elderly on a global scale. Its incidence has skyrocketed with the aging of the population^1^. In particular, Alzheimer’s disease, which accounts for two-thirds of dementia patients, presents a formidable challenge due to the lack of available therapeutic interventions^2^. Vascular dementia represents the second most prevalent subtype of dementia and may contain 15–30% of total dementia cases^3^. In 2017, the World Health Organization (WHO) recommended that all countries launch a global initiative to address dementia from a public health perspective. This initiative has been meticulously crafted to encompass a variety of aspects, including raising awareness of dementia, preventive measures, streamlining the diagnostic process, and enhancing care and treatment modalities for people with dementia^4^. Importantly, early detection of individuals susceptible to dementia has the potential to halt or delay the onset of the disease. Various markers have been used to diagnose the prodromal stage of Alzheimer’s disease more accurately, including (but not limited to) neuropsychological assessment, neuroimaging, and cerebrospinal fluid (CSF) biomarkers^5^.

Peripheral plasma and CSF protein quantification have emerged as auspicious indices for discerning the prodromal pathophysiological cascades precipitating late-onset Alzheimer’s disease. In the annals of Alzheimer’s Disease Neuroimaging Initiative (ADNI), an instructive opus surfaced in 2016, scrutinizing a compendium of 146 plasma and 87 CSF protein biomarkers. Among the pantheon of plasma biological specimens subjected to scrutiny, three salient proteins, namely interferon-γ-inducible protein 10 (IP-10), pregnancy-associated plasma protein A (PAPP-A), and total proinsulin, exhibited the most conspicuous derangements, thereby concomitantly evincing their association with vascular perturbations and the inexorable march of Alzheimer’s disease^6^. Subsequent inquiries into this domain have unveiled corroborative links between vasoregulatory dysfunction and peripheral biomarkers in the context of vascular cognitive impairment. Examples include the discernment of relationships involving Galectin-3^7^ and trigger receptors expressed on myeloid cell 2 (TREM2)^8^. Nevertheless, the complex interactions between serum proinsulin levels and patients’ cognitive domains are still unknown, indicating a need for data clarification in this unexplored area.

Insulin finds its sanctuary in the beta cells of the pancreas, where it is housed in insulin-secreting granules that act as its storehouse. Synthesis of preproinsulin and its subsequent conversion into proinsulin begins. Once meticulously folded and prepared, these newborn substances embark on a journey that culminates in a stay within the Golgi^9^. Proinsulin, the precursor to insulin, is a testament to previous research that firmly established its role as a source of C-peptide, which is secreted by beta cells in equal molar proportions to insulin^10^. Pertinently, the conspicuous elevation of proinsulin secretion relative to insulin and C-peptide levels serves as a notable serological proxy, denoting stress and dysfunction within beta cells—a potential harbinger of impending diabetes mellitus^11^.

Empirical evidence from human and animal studies reveals that insulin has a significant impact on cerebral bioenergetics and displays its role in enhancing synaptic resilience and promoting dendritic spine formation. Furthermore, insulin assumes a pivotal role in the realm of protein homeostasis, exerting influence over the complex processes governing the clearance of amyloid beta peptides and the intricate landscape of tau phosphorylation, both emblematic features of Alzheimer’s disease^12^. Indeed, Alzheimer’s disease has long been associated with insulin, and burgeoning research efforts suggest that patients affected by this cognitive disorder show marked dysregulation of insulin function in peripheral tissues^13^. While the issue of whether insulin is synthesized within the CNS remains a contentious subject, an array of rodent-based studies had provided interesting evidence that insulin mRNA is present within cerebral regions and the release of insulin from GABAergic interneurons and choroid plexus epithelial cells^14,15^.

The hypothesis has been postulated that a plausible common pathogenic mechanism may underlie the interplay among type 2 diabetes, vascular dysregulation, and various forms of dementia, notably Alzheimer’s disease and the vascular dementia subtype. In this context, aberrations in insulin regulation have been implicated as a contributing factor to the pathophysiological processes associated with age-related dementia^16^. A study of the brain of adults with Alzheimer’s disease together with untreated diabetics revealed similar amyloid pathology when compared to Alzheimer’s patients without diabetes. Contrarily, individuals clinically diagnosed with Alzheimer’s disease and comorbid diabetes, who have been subjected to insulin and oral medication regimens, have a markedly relieved amyloid pathology compared to those without Alzheimer’s disease or concomitant diabetes^17,18^. Furthermore, a meta-analysis has furnished evidence of elevated CSF tau concentrations in type 2 diabetes patients in contrast to cognitively intact adults devoid of diabetes^19^. Insulin directly influences the pathogenesis of Alzheimer’s disease through its interaction with Aβ (amyloid-beta) peptides. Insulin exhibits a shielding effect against Aβ synaptoxicity and exerts regulatory dominion over Aβ clearance mechanisms through its modulatory influence upon lipid metabolism and proteases, including the pivotal insulin-degrading enzymes^20^. One study showed a positive correlation between peripheral insulin resistance in Alzheimer’s disease patients and the deposition of Aβ in frontal and temporal brain regions^21^.

Given the notion that chronic peripheral hyperinsulinemia instigates the downregulation of insulin receptors within the blood–brain barrier, culminating in a concomitant reduction in insulin transport to the brain^22^, it is plausible to posit that Alzheimer’s disease patients may experience diminished cerebral insulin levels in the presence of peripheral hyperinsulinemia^23^. The emergence of insulin resistance, as a harbinger of heightened insulin requisites and, ultimately, beta-cell dysfunction, underscores the critical significance of identifying markers of this complex metabolic derangement. In this context, the presence of proinsulin within the peripheral bloodstream has emerged as a salient indicator, signifying the depletion of the intracellular processing enzyme’s cleavage capacity^24^.

The main objective of this study is to closely assess disparities in serum proinsulin levels in two different demographic cohorts stratified by cognitive function—specifically, cohorts encompassing individuals with normal cognition and those beset by cognitive impairment. The overarching aim is to unveil the potential utility of this particular biomarker in elucidating susceptibility to cognitive impairment within the Thai populace.

## Materials and methods

### Enrollment of participants

The prospective enrollment of participants commenced in October 2019 and continued until September 2023. Stringent eligibility criteria were meticulously established, targeting individuals aged 55 years or older, who had sought medical attention at the Dementia Clinic, Neurology Clinic, and Healthy Elderly Clinic situated within the precincts of King Chulalongkorn Memorial Hospital-The Thai Red Cross Society, located in Bangkok, Thailand. A categorical division of patients ensued, predicated upon their scores on the Clinical Dementia Rating Scale (CDR). Patients garnering a CDR score ≥ 0.5 were classified within the ‘cognitive impairment’ cohort, while those displaying a CDR score < 0.5 were categorized within the ‘normal cognition’ cohort. Importantly, individuals under pharmacotherapy for cognitive impairment were included only if they had maintained a stable medication regimen for a minimum of three months prior to enrollment.

Conversely, exclusion criteria were rigorously applied to ensure the homogeneity and integrity of the participant pool. Exclusions encompassed individuals grappling with uncontrolled mental illness, diabetes mellitus with a HbA1C level exceeding 7%, chronic liver disease graded as Child–Pugh Class B, chronic kidney disease characterized by a glomerular filtration rate (GFR) below 30 mL/min, neurosyphilis, chronic HIV infection, active malignancy, and epilepsy that remained poorly controlled.

**Figure 1:**
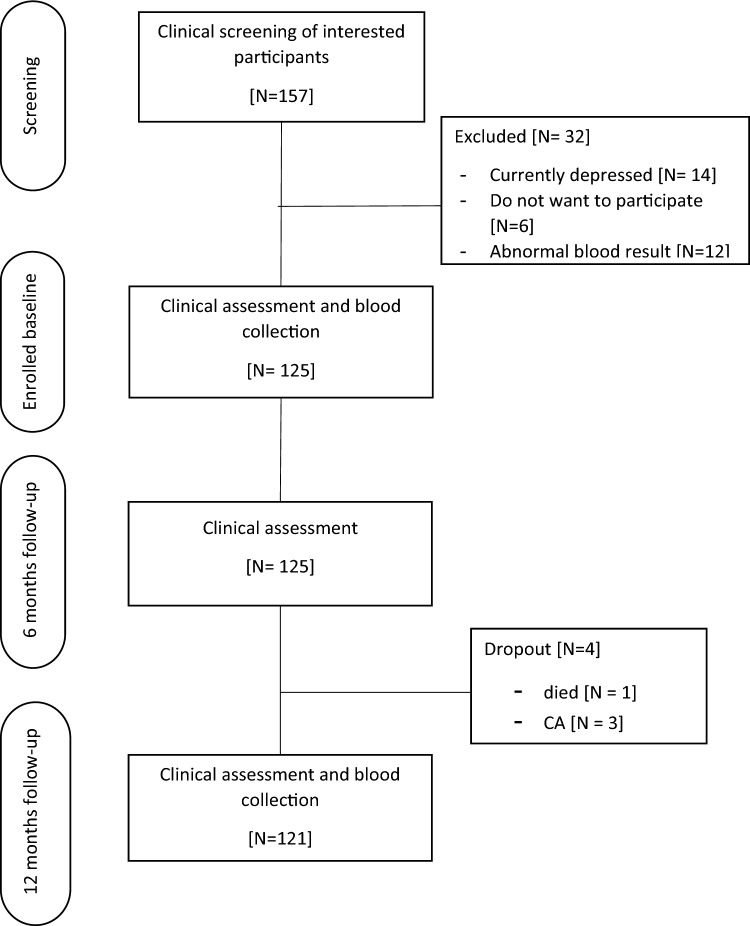
Consort table showing participants.

All participants must have blood drawn for routine testing (complete blood count (CBC), blood urea nitrogen (Bun), creatinine (Cr), blood glucose, lipid profile, etc.), including thyroid function tests. Serum proinsulin was measured at baseline and after 12 months. Neurocognitive tests such as CDR, Mini-mental state examination (MMSE)-Thai 2002, Montreal Cognitive Assessment (MOCA) and Alzheimer’s Disease Assessment Scale (ADAS)-modified were performed every 6 months until the end of the study. The research flow was illustrated in Fig. 1.

### Ethical approval

This study protocol was approved by the Institutional Review Board (IRB) of Chulalongkorn University School of Medicine, Bangkok, Thailand, and was assigned IRB number 317/62. Prior to enrollment, all potential participants or caregivers provided informed consent, thereby confirming voluntary participation in the study. All methods were performed in accordance with the relevant guidelines and regulations by the KCMH ethics committee.

### Statistical analysis

SPSS software version 29 was used to describe and compare epidemiological data between normal and cognitively impaired groups. p-values less than 0.05 were considered statistically significant. This study employed descriptive statistics to elucidate the demographic, clinical characteristics, and laboratory values of individuals categorized into the normal and cognitive impairment groups. Proinsulin data were adjusted by assigning a value of zero in instances where the original measurement fell below 0.75 (the lower limit of detection). Subsequently, the dataset underwent transformation by the addition of 1.00, followed by the application of natural logarithmic functions to achieve a distribution of proinsulin levels approximating normality.

Comparative analyses between the two groups were conducted using independent T-tests and Chi-square tests (or Fisher’s exact test) for the respective variables. Furthermore, to assess temporal changes within each group, paired t-tests were performed by comparing data from two distinct time points. Additionally, a backward-stepwise multiple regression approach was employed to elucidate the association between proinsulin levels and participant group, alongside other pertinent variables (as detailed in the “Results” section). Furthermore, this study explored the relationship between proinsulin and age, as well as proinsulin and cognitive test scores, through Pearson correlation analyses.

### Serum proinsulin detection assay

Serum proinsulin levels for all participants were assessed using the Abcam Human Proinsulin SimpleStep ELISA® Kit (ab242235), following the manufacturer’s prescribed protocols. In brief, following the preparation of all reagents, samples, and standards as per the provided instructions, 50 µL of either the standard or the sample was added to the corresponding wells. Subsequently, 50 µL of the antibody cocktail was introduced into all wells. The incubation was conducted at room temperature for a duration of 1 h, followed by aspiration and triple washing of each well with 350 µL of 1× Wash Buffer. Thereafter, 100 µL of the TMB development solution was added to each well, and a 10-min incubation ensued. Finally, 100 µL of the stop solution was introduced, and optical density (OD) readings were taken at 450 nm. It is noteworthy that the detection limit of this kit is established at 0.75 pmol/L, and the levels below 0.75 pmol/L were set as zero.

## Results

A total of 121 subjects were enlisted as participants in this study. Baseline characteristics, cognitive scores, and underlying conditions are detailed in Table 1. Out of these, 77 individuals were assigned to the normal cognition group, while 44 were allocated to the cognitive impairment group. The demographic composition of the study population revealed a predominant female presence, accounting for 73.55% of the total participants. Notably, the cognitive impairment group exhibited an approximate age difference of 5 years compared to the normal cognition group, and their educational attainment was lower. Within the cognitive impairment group, 12 subjects had received clinical diagnoses of dementia (CDR ≥ 1), with an average onset of symptoms occurring 3 years earlier. Family history of dementia was reported by 14 participants in the normal cognition group and 12 in the cognitive impairment group. No statistically significant disparities were observed in drinking and smoking habits between the two groups. Both groups included participants with underlying diabetes mellitus type 2; however, there were no noteworthy distinctions in the prevalence of diabetes or mean HbA1c levels. Importantly, cognitive test results exhibited significant disparities between the two groups, both at baseline and after a 12-month follow-up period.

**Table 1:** Epidemiological data of both study groups.

Baseline characteristics	Normal cognition group N = 77	Cognitive impairment group N = 44	Total N = 121	p-value
Gender (%, n)				
Female	83.12% (64/77)	56.82% (25/44)	73.55% (89/121)	**0.002**
Male	16.88% (13/77)	43.18% (19/44)	26.45% (32/121)	
Age (year)				
Mean ± SD	64.93 ± 5.25	70.95 ± 7.14	67.12 ± 6.65	** < 0.001**
(Range)	(56–80)	(55–85)	(55–85)	
Highest education (%, n)				
Never study	2.60% (2/77)	5.41% (2/44)	3.31% (4/121)	**0.002**
Elementary school or less	2.60% (2/77)	25.00% (11/44)	10.74% (13/121)	
Middle school	7.79% (6/77)	0	4.96% (6/121)	
High school/vocational ed	10.39% (8/77)	13.64% (6/44)	11.57% (14/121)	
Diploma	9.90% (7/77)	11.36% (5/37)	9.92% (12/121)	
Bachelor’s degree or higher	67.53% (52/77)	45.55% (20/37)	59.50% (72/121)	
BMI (%, n)				
Mean ± SD	23.49 ± 3.67	23.92 ± 3.43	23.65 ± 3.58	0.530
Underlying disease (%, n)				
None	23.38% (18/77)	20.45% (9/44)	20.88% (27/121)	0.767
Diabetes mellitus type 2	10.39% (8/77)	22.73% (10/121)	14.88% (16/121)	0.644
Hypertension	36.36% (28/77)	36.36% (16/44)	36.36% (44/121)	1.000
Dyslipidemia	44.16% (34/77)	52.27% (23/44)	47.11% (57/121)	0.608
Coronary artery disease	1.30% (1/54)	4.54 (2/44)	2.48% (3/121)	0.590
Chronic renal disease	1.30% (1/77)	0	0.83% (1/121)	1.000
Fasting blood sugar				
Mean ± SD	100.09 ± 12.34	99.07 ± 13.05	99.72 ± 12.56	0.669
(Range)	(84–154)	(84–141)	(84–154)	
Hemoglobin A_1_C (%)				
Mean ± SD	5.533 ± 0.45	5.67 ± 0.48	5.58 ± 0.46	0.117
(Range)	(4.6–6.8)	(4.6–7.0)	(4.6–7.0)	
CDR score at baseline (%, n)				
CDR 0	100.00% (77/77)	0	63.64% (77/121)	
CDR 0.5	0	72.73% (32/44)	26.47% (25/121)	
CDR 1	0	18.18% (8/44)	6.61% (8/121)	
CDR 2	0	9.09% (4/44)	3.31% (4/121)	
CDR 3	0	0	0	
MMSE at baseline				
Mean ± SD	28.36 ± 1.32	22.82 ± 6.04	26.35 ± 4.62	** < 0.001**
(Min, Max)	(24, 30)	(6, 30)	(6, 30)	
MMSE at 6 months	[N = 50]	[N = 37]	[N = 116]	
Mean ± SD	28.56 ± 1.31	23.46 ± 6.31	26.67 ± 4.66	** < 0.001**
(Min, Max)	(24, 30)	(2, 29)	(2, 30)	
MMSE at 12 months	[N = 73]	[N = 38]	[N = 111]	
Mean ± SD	28.52 ± 1.25	23.29 ± 6.84	26.73 ± 4.79	** < 0.001**
(Min, Max)	(24, 30)	(1, 30)	(1, 30)	
MOCA at baseline	[N = 74]	[N = 33]	[N = 107]	
Mean ± SD	27.20 ± 1.83	21.42 ± 5.19	25.42 ± 4.25	** < 0.001**
(Min, Max)	(21, 30)	(3, 27)	(3, 30)	
MOCA at 6 months	[N = 70]	[N = 32]	[N = 102]	
Mean ± SD	27.46 ± 1.99	22.12 ± 5.60	25.78 ± 4.30	** < 0.001**
(Min, Max)	(20, 30)	(4, 30)	(4, 30)	
MOCA at 12 months	[N = 72]	[N = 29]	[N = 101]	
Mean ± SD	27.65 ± 1.92	22.45 ± 6.18	26.16 ± 4.35	** < 0.001**
(Min, Max)	(21, 30	(2, 30)	(2, 30)	
ADAS-Cog modified at baseline	[N = 77]	[N = 44]	[N = 121]	
Mean ± SD	6.07 ± 3.36	21.86 ± 15.60	11.81 ± 12.35	** < 0.001**
(Min, Max)	(0.33, 16)	(1.33, 74)	(0.33, 74)	
ADAS-Cog modified at 6 months	[N = 73]	[N = 42]	[N = 115]	
Mean ± SD	5.53 ± 3.35	22.16 ± 17.37	11.60 ± 13.39	** < 0.001**
(Min, Max)	(0.67, 15)	(2.67, 75)	(0.67, 75)	
ADAS-Cog modified at 12 months	[N = 74]	[N = 38]	[N = 112]	
Mean ± SD	5.69 ± 3.24	22.21 ± 17.28	11.30 ± 13.19	** < 0.001**
(Min, Max)	(0.33, 18)	(1.67, 82)	(0.33, 82)	

Significant values are in bold.

### Serum proinsulin level

In the cohort characterized by normal cognitive function, the mean baseline and 12-month serum proinsulin levels were 6.10 (± 8.43) and 7.90 (± 10.10) pmol/L, respectively, exhibiting a marginally elevated level with a non-significant p-value of 0.159. In the cognitive impairment cohort, the mean baseline serum proinsulin level stood at 10.35 (± 10.98) pmol/L, while the corresponding 12-month measurement was 8.37 (± 6.94) pmol/L, yielding a p-value of 0.261. (Table 2).

**Table 2:** Level of serum proinsulin in normal cognition vs. cognitive impairment groups.

Proinsulin level	Normal cognition group N = 77	Cognitive impairment group N = 44	p-value
At baseline			
Mean proinsulin level (pmol/L) (± SD)	6.095 (± 8.434)	10.348 (± 10.976)	**0.019**
At 12th month			
Mean Proinsulin level (pmol/L) (± SD)	7.901 (± 10.097)	8.372 (± 6.942)	0.784
Average			
Mean Proinsulin level (pmol/L) (± SD)	6.998 (± 7.449)	9.630 (± 7.164)	0.092

Significant values are in bold.

### Baseline proinsulin in the two groups

In the present study, a backward-stepwise regression analysis was conducted, employing transformed baseline proinsulin levels as the dependent variable and a set of independent variables including age, gender, education, history of dyslipidemia (0/1), history of chronic renal disease (0/1), history of diabetes (0/1), count of other medical conditions, BMI, fasting plasma glucose, and HbA1C. The results of the regression analysis (Table 3) demonstrated a statistically significant association between the participant group and BMI with the baseline proinsulin levels. Furthermore, a trend of association was observed for HbA1c and diabetes. In contrast, gender, educational attainment, the history of chronic renal disease, and fasting plasma glucose exhibited no statistically significant associations with the baseline proinsulin levels and thus were excluded from the stepwise selection process.

**Table 3:** Result of regression model of baseline proinsulin and the retained demographic and clinical variables.

	Coefficient	SE	p-value	95% confidence interval	Adjusted R-squared
Baseline proinsulin					0.134
Group	0.385	0.193	**0.048**	0.003, 0.767	
BMI	0.067	0.026	**0.012**	0.015, 0.119	
HbA1c	0.391	0.203	0.056	− 0.011, 0.792	
Diabetes	0.462	0.262	0.081	− 0.057, 0.980	
Medical conditions	0.212	0.151	0.164	− 0.088, 0.511	
Dyslipidemia	− 0.240	0.183	0.193	− 0.603, 0.123	
Constant	− 2.340	1.371	0.091	− 5.057, 0.376	

Significant values are in bold.

### Serum proinsulin and age

**Figure 2:**
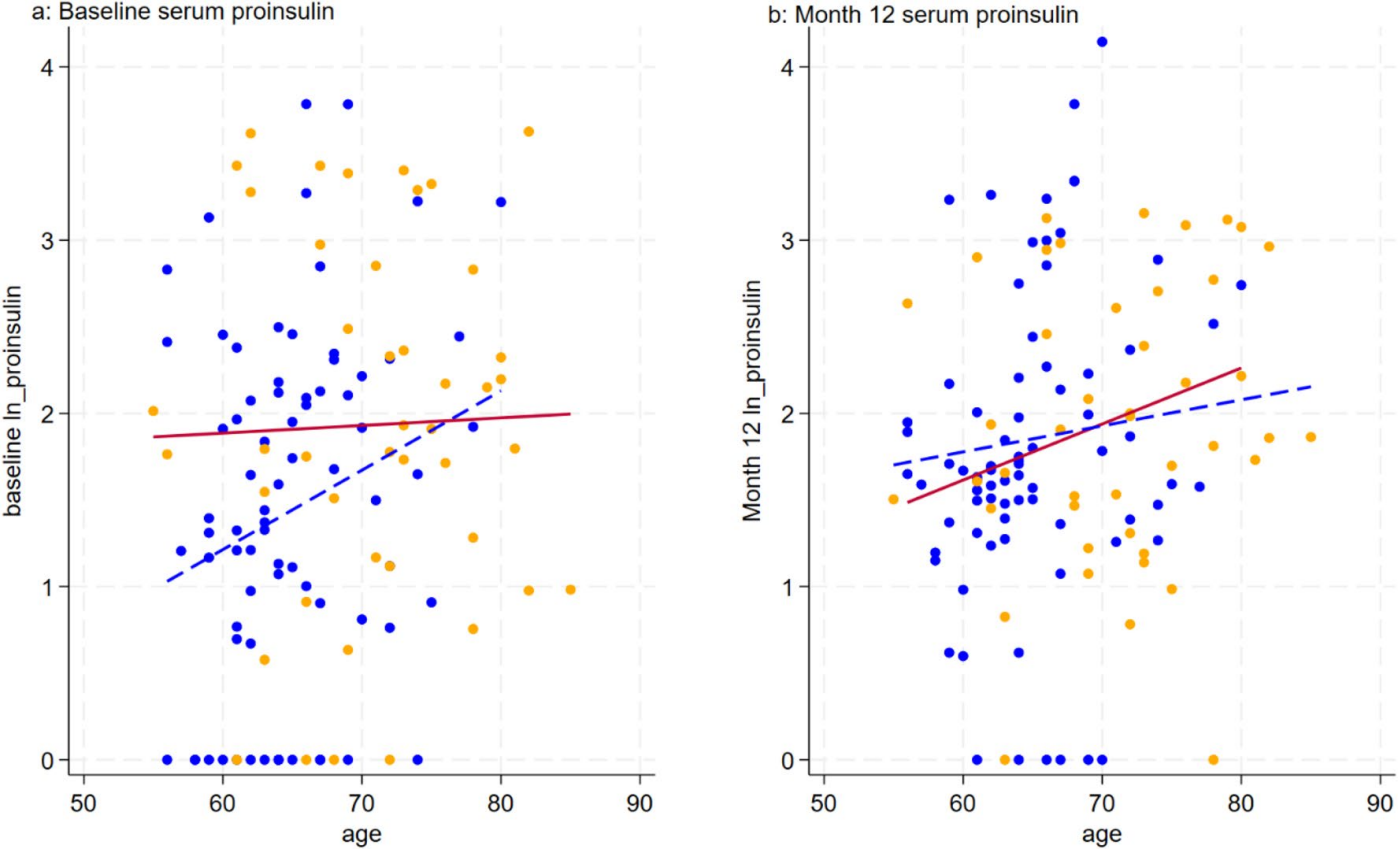
Scatter plot and line plot between serum proinsulin and age in normal and cognitive impairment group. Blue dot = normal cognition group, Yellow dot = cognitive impairment group.

Divergent correlations between proinsulin and age were observed within the cognitive impairment and normal cognitive function groups (Fig. 2). Differences in the correlation between proinsulin and age were observed within the cognitive impairment group (r = 0.030, p-value = 0.849) and the normal group (r = 0.234, p-value = 0.041). Within the normal group, a positive correlation was evident, indicating that baseline proinsulin levels increased with age. Conversely, in the cognitive impairment group, there was no significant change in baseline proinsulin levels with increasing age.

Comparable outcomes emerged upon reevaluating proinsulin levels after 12 months. In the cognitive impairment group, the correlation between proinsulin at month 12 was non-significant (r = 0.131, p-value = 0.397). Conversely, in the normal group, the correlation approached statistical significance (r = 0.191, p-value = 0.096).

Moreover, our analysis revealed disparate changes in proinsulin levels between the two participant groups. The mean change in proinsulin levels from baseline to month 12 was − 1.975 (SD = 11.492) in the cognitive impairment group, while it was 1.806 (SD = 11.146) in the normal group. Although this disparity demonstrated only a trend towards statistical significance (t-test = 1.775, p-value = 0.079), it suggests a noteworthy divergence in proinsulin dynamics between the cognitive impairment and normal groups.

### Proinsulin and cognitive test score

A negative association was observed between proinsulin levels and cognitive performance (Table 4). In the overall participant cohort, correlation analysis revealed a significant association between baseline proinsulin levels and a decline in cognitive scores on the ADAS-cog, although statistical significance was not reached for the MMSE and the MOCA. However, no statistically significant correlations were found between baseline proinsulin levels and cognitive test scores at month 12, nor between proinsulin levels at month 12 and cognitive test scores at month 12.

**Table 4:** Correlation coefficient and p-value (in brackets) between proinsulin and MMSE, MOCA and ADAS at baseline and month 12 of all participants and by groups.

	Total		Cognitive impairment group		Normal group	
	Baseline proinsulin	Month 12 proinsulin	Baseline proinsulin	Month 12 proinsulin	Baseline proinsulin	Month 12 proinsulin
At baseline						
MMSE	− 0.171 (0.062)		− 0.013 (0.933)		− **0.250 (0.028)**	
MOCA	− 0.157(0.108)		0.089 (0.621)		− **0.364 (0.001)**	
ADAS-cog	**0.256 (0.009)**		0.039 (0.804)		**0.554 (< 0.001)**	
At month 12						
MMSE	− 0.069 (0.473)	− 0.026 (0.786)	0.080 (0.634)	− 0.035 (0.837)	0.072 (0.548)	− 0.046 (0.702)
MOCA	− 0.064 (0.525)	− 0.127 (0.201)	0.158 (0.413)	0.010 (0.718)	− **0.307 (0.009)**	− 0.079 (0.508)
ADAS-cog	0.162 (0.089)	0.076 (0.429)	− 0.026 (0.879)	0.060 (0.719)	**0.290 (0.012)**	0.046 (0.696)

Significant values are in bold.

Subgroup analysis within the cognitive impairment group revealed no significant correlations between baseline proinsulin levels and cognitive test scores at baseline or month 12, nor between proinsulin levels at month 12 and cognitive test scores at month 12. In contrast, the results differed for the normal group, where significant correlations were identified between baseline proinsulin levels and all baseline cognitive test scores. Additionally, correlations were observed between baseline proinsulin levels and cognitive test scores at month 12 for the MOCA and ADAS-cog. However, no statistically significant correlation was found between proinsulin levels at month 12 and cognitive test scores at month 12. Furthermore, there were 10 participants in the cognitively impaired group with a CDR < 0.5 at 12 months and 4 participants in the normal group with a CDR ≥ 0.5 at 12 months. These may influence the assessment of serum proinsulin and cognitive tests in these subgroups.

## Discussion

In our study, participants within the cognitive impairment group exhibited notably elevated serum proinsulin levels at the baseline of recruitment. Despite the absence of discernible disparities in glycemic control or underlying diabetic conditions, the baseline serum proinsulin levels were notably higher in the cognitive impairment cohort compared to the normal cognitive function group (as detailed in Table 3). However, at the 12-month mark, there was no statistically significant divergence in serum proinsulin levels between these two cohorts. Nonetheless, there was merely a discernible trend suggesting that overall proinsulin levels in patients with cognitive impairment were slightly elevated compared to those with normal cognitive function. Furthermore, our investigation identified cognitive status, age, and BMI as potential factors associated with variations in baseline proinsulin levels. Specifically, within the normal cognitive group, an association was observed between increasing age and elevated serum proinsulin levels, consistent with findings from several prior studies^25,26^. Intriguingly, this correlation was notably absent within our cognitively impaired groups.

An interesting observation emerged from our study, where the normal cognition group exhibited a higher proportion of females compared to the cognitive impairment group. Nevertheless, our regression analysis incorporating baseline proinsulin levels and clinical variables did not reveal any significant associations involving gender and proinsulin levels in our study cohort.

Our study represents the inaugural investigation to unveil a correlation between serum proinsulin levels and cognitive status scores. In summary, a heightened baseline proinsulin level appears to be associated with diminished cognitive performance, particularly within the normal cognitive group. Additionally, baseline proinsulin levels may exhibit a connection with cognitive performance at the 12-month mark in the normal group. However, no significant correlation between proinsulin levels and cognitive performance was discerned within the cognitive impairment group. These findings lend credence to the hypothesis positing the significance of insulin in maintaining brain health and suggest a potential role for peripheral and central insulin dysregulation in the development of cognitive impairment. Consequently, enhancing insulin availability and sensitivity within the central nervous system may hold promise in mitigating or postponing the onset of Alzheimer’s disease and related disorders^12^.

Emerging evidence suggests the potential efficacy of certain antidiabetic medications in the treatment of Alzheimer’s disease, both in human subjects and animal models^27^. Among these, the most promising candidate is treatment with glucagon-like peptide-1 receptor agonists (GLP1-RA). GLP-1, a well-established neurotransmitter, is expressed in various brain regions, including the striatum, nucleus accumbens, and hippocampus, through its corresponding receptors^28^. In a comprehensive study, Nørgaard et al. assessed GLP-1 RA exposure and subsequent dementia diagnoses among individuals with type 2 diabetes, drawing data from three randomized, double-blind, placebo-controlled trials encompassing over 130,000 participants. Their findings demonstrated that GLP-1 RA treatment could significantly reduce the incidence of dementia in individuals with type 2 diabetes^29^. Consequently, the evaluation of serum proinsulin levels may offer a valuable tool for identifying non-diabetic individuals with cognitive impairment who could benefit from emerging Alzheimer’s treatments associated with the insulin pathway, such as GLP1-RA.

This study is not devoid of limitations. Firstly, the sample size was relatively modest, potentially constraining the depth of insights gleaned from proinsulin as a biomarker for cognitive impairment. Future investigations should encompass a larger cohort of participants to yield a more comprehensive dataset. Secondly, a subset of participants exhibited serum proinsulin levels falling below the detection limit. Employing highly sensitive digital immunoassay techniques may facilitate a more precise determination of serum proinsulin levels in such cases. Thirdly, our study delineated participant groups solely based on baseline CDR scores, and considering additional biomarkers, such as amyloid or tau PET scans, may enhance diagnostic accuracy. Fourthly, assessing serum proinsulin levels at a third time point may provide further insights into the trends related to cognitive impairment and proinsulin levels.

## Conclusion

Considering the intricate relationship between insulin resistance and the pathogenesis of Alzheimer’s disease, it is plausible that serum biomarkers associated with insulin metabolism might display irregularities in individuals affected by dementia. In this study, we have examined serum proinsulin levels in individuals with normal cognitive function and those suffering from cognitive impairment, revealing a significant disparity between the two groups. This finding offers potential implications for the identification of non-diabetic patients eligible for novel Alzheimer’s drug therapy that related to insulin pathway. Additionally, serum proinsulin levels hold promise as a potential biological marker for detecting cognitive decline in the elderly population.

## Data Availability

The datasets used and/or analyze during the current study are available from the corresponding authors on reasonable request.
